# Effectiveness of low dose computed tomography to detect fractures in paediatric suspected physical abuse: a systematic review

**DOI:** 10.1007/s00414-024-03214-2

**Published:** 2024-04-15

**Authors:** Edel Doyle, Lyndal Bugeja, Matthew R. Dimmock, Kam L. Lee, Jessica Ng, Richard B. Bassed

**Affiliations:** 1 Department of Forensic Medicine, Monash University, 65 Kavanagh Street, Southbank, VIC 3004 Australia; 2 Department of Medical Imaging and Radiation Sciences, Monash University, Melbourne, VIC Australia; 3 Australian Radiation Protection and Nuclear Safety Agency, Yallambie, VIC Australia; 4 Cabrini Health, Melbourne, VIC Australia; 5 Victorian Institute of Forensic Medicine, Academic Programs, Melbourne, VIC Australia; 6 School of Allied Health Professions, Keele University, Keele, UK

**Keywords:** Computed tomography (CT), Child, Non-accidental injury (NAI), Physical abuse, Inflicted injury, Diagnostic accuracy test

## Abstract

**Purpose:**

The skeletal survey X-ray series is the current ‘gold standard’ when investigating suspected physical abuse (SPA) of children, in addition to a non-contrast computed tomography (CT) brain scan. This systematic literature review synthesised findings of published research to determine if low dose computed tomography (LDCT) could detect subtle fractures and therefore replace the skeletal survey X-ray series in the investigation of SPA in children aged under 3 years.

**Methods:**

Five electronic databases and grey literature were systematically searched from their inception to 28 April 2022. Primary studies were included where the population comprised paediatric patients up to 16 years and LDCT was used to detect fractures associated with SPA. Studies involving imaging investigations of the head, standard dose CT examinations or accidental trauma were excluded.

**Results:**

Three studies met the inclusion criteria, all of which were case series. These studies did not report many of the criteria required to compare the accuracy of LDCT to X-ray, i.e. they did not meet the criteria for a diagnostic accuracy test. Therefore, it is difficult to conclude from the case series if LDCT is accurate enough to replace X-rays.

**Conclusion:**

Due to the gap in current literature, a phantom study and subsequent post-mortem CT study are recommended as the primary investigative methods to assess the ability of low-dose CT to identify the subtle fractures associated with SPA and to calculate how low the achievable CT dose can be.

**Supplementary Information:**

The online version contains supplementary material available at 10.1007/s00414-024-03214-2.

## Introduction

Suspected physical abuse (SPA) or non-accidental injury (NAI) affects vulnerable members of our society, particularly children. According to the most recent estimates, 50% of children aged 2–17 years globally experienced some form of violence in the previous year [1]. The early detection of child abuse is crucial to reduce the risk of escalation and mortality [2]. Medical imaging is one method of detecting injuries that have resulted from SPA; this is particularly important for children under the age of 2 years who are not able to verbally communicate and thereby rely on the healthcare system to advocate for them. However, young children are particularly sensitive to the harmful effects of radiation meaning computed tomography (CT) is not routinely used. Image quality is proportional to radiation dose which is an important consideration when imaging children who are considered to be more radiosensitive than adults; primarily due to the longer latency period in which to develop cancer over their lifetime. Therefore, special efforts are made to optimise radiographic examinations using X-ray projection imaging to keep the radiation dose “as low as reasonably achievable” (ALARA) [3]. Low dose CT (LDCT) protocols have been implemented by vendors for specific clinical indications, e.g. lung nodules, multiple myeloma. It is acknowledged that there is no internationally accepted definition of LDCT, although the medical imaging industry generally accepts that any dose lower than 1 mSv can be described as “low dose.” Therefore, this definition was adopted for the study.

Recommendations published by international professional bodies guide selection of which radiographic projections should be acquired to best demonstrate the subtle fractures associated with SPA in conjunction with a non-contrast CT scan of the brain [4–7]. Collectively, this series of X-rays is referred to as a radiographic skeletal survey. The recommended protocols include a range of 11–33 X-ray projections with additional projections recommended to provide further detail [5, 8]. The technical challenges encountered when acquiring this series of radiographs on a living child have been identified as: the size of the child, the diverging X-ray beam and the level of co-operation from the child [6]. Considerations also include the number of X-ray projections required and therefore, the time taken to acquire these images. The Royal College of Radiologists (RCR) note that the skeletal survey can be distressing for the children, their family and staff involved particularly due to immobilisation requirements [5]. Younger children usually need to be immobilised to reduce motion artefacts. Repeat images are sometimes required and increases the cumulative radiation dose to the child [9, 10]. Given the medicolegal indications for this type of imaging, it should also be considered that one of the child’s carers who may be assisting with immobilisation could be the suspected abuser. Together, these factors can contribute to a very challenging environment for the radiographers who should be working in pairs to document the entire imaging examination, whilst acquiring high quality diagnostic images for the radiologist [11].

Metaphyseal (corner or bucket-handle) fractures are indicators of SPA, particularly multiple fractures of varying ages and/or in conjunction with rib fractures or a head injury [12]. As metaphyseal fractures are caused by a twisting action, the mechanisms of injury associated with accidental trauma in this age group are quite different. Importantly, metaphyseal fractures are subtle and can be difficult to detect. However, as the spatial resolution of X-ray is higher than CT (3–5 line pairs per millimetre compared to 0.7–1.2) [13, 14], the diagnostic threshold for visualising metaphyseal fractures using low dose CT (LDCT) has not yet been established and may be a limiting factor to their use.

Following the publication of a national protocol in New Zealand in 2015, it was estimated that a child under 3 years of age would receive an effective radiation dose of approximately 0.2 milliSieverts (mSv) for a standard series of approximately 17 X-ray projections [6, 15]. A phantom study in Australia based on the RCR guidelines, including 31 projections, estimated an effective radiation dose of 0.09 mSv for a two-year-old child [16]. Given the number of projections required to complete a Skeletal Survey X-ray series and a potentially distressed child and/or carers [17], this may take a significant amount of time to achieve in a clinical setting depending on the experience of the radiographers. A whole-body CT scan should take a few minutes to acquire with the benefit that the volumetric data can be reconstructed retrospectively in any plane, thereby eliminating the need for additional imaging to be acquired to assist with the initial diagnosis. A LDCT skeletal survey would be significantly less traumatic for the child compared to being moved into different positions to accommodate 11–33 X-ray projections. In other settings, the use of LDCT skeletal surveys has already replaced radiographic skeletal surveys in adults e.g. multiple myeloma [18]. In addition, a recent case series of 10 infants aged up to 8 months reported that the effective radiation dose for LDCT skeletal surveys for SPA ranged from 0.73 to 1.46 mSv [19]. There is no intention to replace the existing requirement for a non-contrast CT Brain scan which is performed to look for acute haemorrhage/s suggestive of abusive head trauma and requires a higher radiation dose than for bones which have a higher inherent contrast resolution. A CT brain scan is estimated to provide an effective radiation dose of 2.49 mSv [15]. Therefore, the cumulative radiation dose for a child having a CT brain and a LDCT skeletal survey would be 2.7 mSv. It is acknowledged that whilst CT scan protocols for post-mortem imaging of children have been published, radiation doses have not been calculated because radiation dose is not a consideration in the deceased so image quality can be prioritised [5].

A systematised literature review established that no peer-reviewed original research has been performed to determine whether a LDCT skeletal survey should be used instead of a radiographic skeletal survey in the investigation of paediatric SPA [20]. Therefore, in this paper, the eligibility criteria have been expanded to include LDCT of any body part in the detection of fractures associated with paediatric SPA. This systematic literature review seeks to identify and synthesise the scientific research evidence on whether LDCT could replace X-rays for the investigation of SPA in children aged under 3 years.

## Methods

This systematic review was performed in accordance with the Preferred Reporting Items for Systematic Reviews and Meta-Analyses (PRISMA) 2020 [21].

### Eligibility criteria

Studies were included in the review where they met the following criteria:

- the research design was a case report, case series, case control study, cohort study, or randomised controlled trials
- the study population were children aged under 3 years or younger;
- the study compared LDCT to a standard-dose CT or an X-ray to detect a fracture;
- the fracture (e.g. subtle metaphyseal fractures, long bone fractures, rib fractures) was thought to be caused by SPA; and
- the study was published after 2011 as LDCT technology was not available prior to then.

### Information sources

The information sources for this review comprised the following five electronic databases: the Cochrane Library, Embase (via Ovid), MEDLINE(R) (via Ovid), Scopus (via Elsevier) and Web of Science Core Collection, accessed via Monash University library. Grey literature databases and internet search engines including Google, DuckDuckGo and Millionshort were searched, as well as targeted websites (Supplemental Information). A bibliographic review of articles that met the inclusion criteria were also reviewed to identify any studies that may not have been indexed in the databases searched.

### Search strategy

Advice was sought and support provided by university and medical librarians to identify and test key words and indexed terms (e.g. Medical Subject Heading) to develop a search strategy. The search strategy for each database and the indexed terms used to search each database are shown in the Supplemental Information. The final searches were re-run on 28 April 2022.

### Selection process

The results from each of the database searches were imported into Covidence and duplicates were removed [22]. Two reviewers independently [ED and JN] screened the titles and abstracts against the eligibility criteria. Conflicts were independently adjudicated by a third reviewer [RB]. The full texts of the publications that met the inclusion criteria were imported and independently reviewed by the first two reviewers [ED and JN] and conflicts were resolved by consensus.

### Data collection process

Data extraction from included studies was performed independently by two reviewers [ED and JN] [23]. The following information was recorded from each study using the data extraction template function in Covidence: study design; study population (i.e. number of patients); patient age; body part scanned; CT scanned used; CT protocol used (i.e. scan parameters such as kV, mA (fixed or automatic)) exposure time, algorithm; and outcome measures (i.e. accuracy measures, radiation doses).

### Study risk of bias assessment

A risk of bias assessment was independently performed by two reviewers using the Joannna Briggs Institute (JBI) Critical Appraisal Checklist for Case Series [24]. The JBI checklist assesses the trustworthiness, relevance and results of included studies and enables an overall rating to be assigned to each study. “Yes” was scored as 0 and “unclear/no” was scored as 1. The number of criteria met was tallied to form the quality score for each study. A final quality rating of “low,” “moderate,” or “high” quality was given to each study according to the overall score. The following scoring parameters were used: JBI Checklist for Case Series Studies (score out of 9); Low score 0–3 represented higher quality, Moderate 4–6 and a High score of 7–9 represented poorer quality. Each study was appraised independently by two reviewers [ED and JN] with discordance adjudicated by a third reviewer [LB].

The same method was repeated using a second risk assessment tool, QUADAS-2 [25].

### Effect measures

The primary outcome measure was image quality, which was assessed by extracting sensitivity, specificity, positive predictive value, negative predictive value, confidence intervals, inter- and intra-observer reliability or Receiver Operating Characteristic when comparing X-ray to LDCT. The secondary outcome was radiation dose which was assessed using Kerma Area Product (KAP) for X-rays and volumetric computed tomography index (CTDI_vol_) with dose length product (DLP) for CT. Radiation dose assessment between these two modalities was calculated and reported as Effective Dose to enable comparison.

### Synthesis methods

The study characteristics and outcomes were presented in a standardised manner for each of the included studies in a tabular format to enable similarities and differences to be identified. The study characteristics included: study design; study population (i.e. number of patients); patient age; body part scanned; CT scanned used; CT protocol used (i.e. scan parameters such as kV, mA (fixed or automatic)) exposure time, algorithm. The study outcome synthesis comprised:

- Efficacy of low dose CT scans compared to X-ray (or standard dose CT)
- Image Quality assessment (low dose CT versus X-ray/standard dose CT)
- Radiation dose comparison (low dose CT versus X-ray/standard dose CT)

### Reporting bias assessment

Where results were missing, they were documented as “not reported” (NR).

### Certainty assessment

None of the three studies estimated diagnostic accuracy and its precision.

## Results

### Study selection

**Fig. 1:**
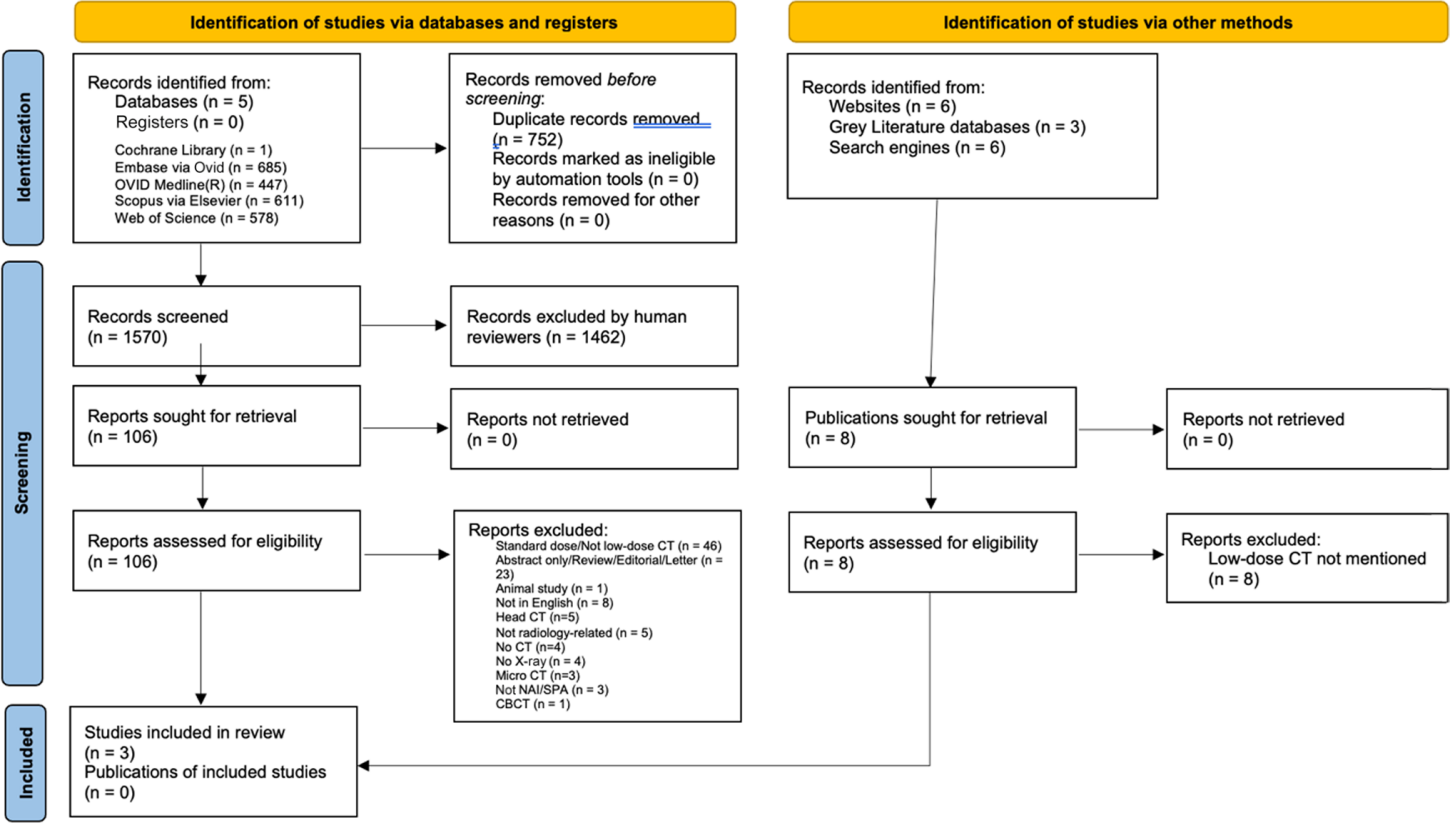
PRISMA 2020 flow diagramr [21]

Database searches including grey literature sources yielded 2,337 records. 752 records were removed by Covidence prior to screening because they were duplicates. 1,570 records were excluded during screening as they did not meet the inclusion criteria. 106 full-text articles were assessed, 103 of which were excluded with the reasons shown in Fig. 1.

Three articles met the inclusion criteria, as shown in Fig. 1 [19, 26, 27].

### Study characteristics

The three studies were all case series design, published between 2012 and 2021 from two different countries and performed in a hospital (Table 1). Three studies reported study duration which ranged from 14 to 49 months. Study participants comprised three human studies involving 30 children whose age ranged from 3 weeks to 11 months.

**Table 1:** Summary of the study and baseline characteristics of the included studies

Study characteristics				
Variable		Sanchez (2015) [26]	Sanchez (2018) [27]	Lawson (2022) [19]
Country		USA	USA	Australia
Study Design		Case series	Case series	Case series
Study Period and Duration (Months)		Jan 2013 to Feb 2014 (14 months)	Jan 2008 to May 2012 (4 years & 1 month = 49 months)	June 2019 to Sep 2020 (16 months)
Setting		Hospital	Hospital	Hospital
Method of Recruitment		Admitted patients	Clinically diagnosed cases of NAI	Too unstable OR parental choice
Number of Participants		4	16	10
Participant Age		0.08–0.33 years (1–4 months)	0.08–0.9 years (1–11 months)	0–0.66 years (3 weeks to 8 months)
Body part		Chest	Chest	Orbits to toes (+ routine dose non-contrast CT Brain)
CT scanner		GE LightSpeed VCT	NR	GE Discovery 750HD
Number of slices		64	NR	64
Quality Assessment (JBI ‘total’ scores)		2	3	0
Baseline characteristics				
X-ray	Number of Studies	Sanchez (2015) [26]	Sanchez (2018) [27]	Lawson (2022) [19]
Male (n)	2	3	7	NR
Female (n)	2	1	9	NR
Total number of participants	20	4	16	NR
Low Dose CT				
Male (n)	3	3	7	7
Female (n)	3	1	9	3
Total number of participants	30	4	16	10

where *NR* not reported, *NA* not applicable

Two of the studies only scanned the chest, whereas the third scanned from the orbits to the toes. Two of the studies reported that a 64-slice CT scanner was used, which were GE LightSpeed VCT and GE Discovery 750HD.

Baseline participant characteristics were reported for the X-ray image modality and LDCT in two of the three studies (Table 1). For the X-ray image modality, this comprised 10 male and 10 female participants (2 studies). For the LDCT image modality there were 17 male participants and 13 female participants across the 3 studies.

The scan parameters used to acquire the LDCT scans ranged from 80 to 100 kV and all three studies appear to have used fixed mA ranging from 15 to 50 with a rotation time of 0.4 or 0.5 seconds (Table 2). Pitch ranged from 0.9 to 1.5. Collimation was reported by two of the studies as 64 rows of 0.6 mm detectors. Only one study reported CTDI_vol_ ranging from 0.31 to 0.64 mGy and used a 32 cm phantom (current best practice) [19], rather than 16 cm which was traditionally used for paediatrics [28]. The slice thickness of reconstructed images was only reported for one of the studies and was 1.25 or 5 mm with the increment not reported. None of the studies reported the algorithm applied to the raw data, although two studies noted that 20% iterative reconstruction was applied [26, 27].

**Table 2:** Summary of the LDCT protocols

Scan Parameters	Number of Studies	Sanchez (2015) [26]	Sanchez (2018) [27]	Lawson (2022) [19]
kV_p_	4	80–100	100	80
mA	4	15	15	20–50
Rotation Time (s)	4	0.5	0.5	0.4
Automatic dose modulation	0	NR	NR	NR
Pitch	4	0.9, 1.5	1	0.97
Focal spot size	0	NR	NR	NR
Collimation	3	64 × 0.6 mm	NR	64 × 0.6 mm
Phantom size (cm)	2	NR	NR	32
CTDI_vol_ (mGy)	1	NR	NR	0.31–0.64
Reconstruction	2	1.25 mm AX 3x5mm COR & SAG	NR	NR
Algorithm	1	20% ASIR	20% ASIR	NR

where *NR* not reported; *AX* axial; *COR* coronal; *SAG* saggital

Diagnostic Accuracy has been collated in Table 3. Only one study attempted to report the total accuracy of fracture detection on X-rays and quoted a positive predictive value (PPV) of 83% [27]. None of the LDCT studies reported total accuracy of fracture detection nor inter-observer reliability.

**Table 3:** Summary of Diagnostic Accuracy assessment

X-ray	Number of Studies	Sanchez (2015) [26]	Sanchez (2018) [27]	Lawson (2022) [19]
Total accuracy	1	NR	“On the initial survey, only 87 of 105 fractures (83%) were seen. Eleven (11/18) of these fractures were identified on the chest CT scans of 5 patients, whereas 7 (7/18) fractures were identified on the follow-up skeletal surveys in patients who did not have chest CT.”	NR
Sensitivity	0	NR	NR	NR
Specificity	0	NR	NR	NR
Positive Predictive Value	1	NR	83%	NR
Negative Predictive Value	0	NR	NR	NR
Confidence Interval	0	NR	NR	NR
Inter-Observer Reliability	0	NR	NR	NR
Intra-Observer Reliability	0	NR	NR	NR
Receiver operator Curve	0	NR	NR	NR
LDCT				
Total accuracy	0	NR	NR	NR
Sensitivity	0	NR	NR	NR
Specificity	0	NR	NR	NR
Positive Predictive Value	0	NR	NR	NR
Negative Predictive Value	0	NR	NR	NR
Confidence Interval	0	NR	NR	NR
Inter-Observer Reliability	0	NR	NR	NR
Intra-Observer Reliability	0	NR	NR	NR
Receiver operator Curve	0	NR	NR	NR

where *NR* not reported

A comparison of the effective radiation doses between the X-ray and LDCT was reported in all three studies (Table 4). The effective doses reported for the X-rays range from 0.002 to 0.35 mSv compared to the LDCT scans which range from < 0.45 to 1.46 mSv. The difference in body parts scanned are noted with two only scanning the chest [26, 27] and the third scanned from vertex or orbits to toes [19].

**Table 4:** Summary of comparison of radiation doses

X-ray	Number of Studies	Sanchez (2015) [26]	Sanchez (2018) [27]	Lawson (2022) [19]
KAP (cGy·cm^2^)	0	NR	NR	NR
Effective Dose (mSv)	3	0.19–0.35	0.35	0.002–0.12
LDCT				
CTDI_vol_ (mGy)	1	NR	NR	0.31–0.64
DLP (mGy·cm)	1	NR	NR	17.63–35.32
Effective Dose (mSv)	3	0.45–1.13	0.48	Vertex to toes: 0.73–1.02 Orbit to toes: 0.86–1.46 Brain: 0.39–0.62

where *NR* not reported

None of the authors directly addressed the validity of their study so it can only be assumed that the images produced were diagnostic.

Reliability refers to measurement consistency. If the same result can be consistently achieved by using the same methods under the same circumstances, the measurement is considered reliable, e.g. inter-rater reliability. Two observers participated in one of the studies [19]. Two of the studies did not report how many observers were involved [26, 27]. Inter-observer reliability was not reported for any of the three studies (Table 5).

**Table 5:** Summary of validity of included studies

Number of Studies	Sanchez (2015) [26]	Sanchez (2018) [27]	Lawson (2022) [19]
0	Low	Low	NR

where *NR* not reported

### Risk of bias in studies

**Fig. 2:**
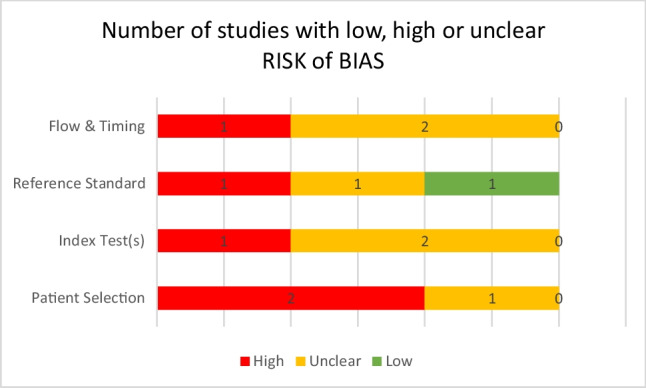
Number of studies with low, unclear or high risk of bias according to the QUADAS-2 tool [25]

Table 1 presents the total score from the JBI assessment of quality. The use of the QUADAS-2 tool validated the previous results (Fig. 2). Therefore, it can be concluded that overall, the methodological quality of the three studies was poor, with only one study having a low risk of bias in relation to the Reference Standard.

### Results of syntheses

None of the three articles were a Diagnostic Test Accuracy (DTA) study, so it was not possible to do a meta-analysis. Therefore, three outcomes were reported upon.

#### Outcome 1: accuracy of low dose CT scans compared to X-ray (or standard dose CT)

One study reported two measures for Diagnostic Accuracy of X-rays [27], but none of the three studies compared the accuracy of X-rays to LDCT.

#### Outcome 2: image quality assessment (low dose CT versus X-ray/standard dose CT)

None of the studies considered the effect of radiation dose on image quality. Lawson et al. specifically stated that injuries visualised on post-mortem CT were not identified on their LDCT scans whilst the same children were still alive but the reasons for missing these injuries were not reported [19].

#### Outcome 3: radiation dose comparison (low dose CT versus X-ray/standard dose CT)

All three studies reported on kV_p_, mA, rotation time and pitch but none noted if automatic tube current modulation was used. Two studies reported the number of slices and collimation used [19, 26]. One study reported the reconstructed slice thickness which ranged from 1.25 to 3 mm [26]. Only one study reported the phantom size used and the CTDI_vol_ [19]. None of the studies reported the algorithms used. The effective radiation doses range from 0.45 to 1.46 mSv for the CT scans compared to 0.002 to 0.35 mSv for the X-rays.

The lowest kV_p_ used in the scan protocols across the three studies was 80 kV_p_ but a wider range of mA values (15 to 50) were utilised so there was no consensus on the lowest exposure factors that produced diagnostic images, although two of the three studies used a rotation time of 0.4 seconds whilst the third used 0.5 seconds. Pitch ranged from 0.9 to 1.5. The two studies that reported the number of slices and collimation used were 64 × 0.6 mm. The only study that reported reconstructed slice thicknesses was 1.25 mm for axial images and 3 mm slice thickness with 5 mm increments for coronal and sagittal images. One study reported the phantom size used (32 cm), and was also the only study to report CTDI_vol_ which is now required when publishing for comparison of CT doses between vendors. The algorithm used was not reported for any of the studies. Neither did any of the studies mentioned the use of automatic tube current modulation so it is assumed that all used fixed mA technique. Focal spot size was not reported by any of the studies. The inconsistency in scan details reported makes replication of the studies almost impossible.

Whilst the effective radiation doses are reported in Table 4, none of the three studies considered the effect of radiation dose on image quality.

### Reporting biases

Publication bias may be present in all three studies, as all conclude that LDCT is achievable and diagnostic in the detection of subtle fractures associated with SPA. There is evidence of outcome reporting bias in one study, as there was no reference standard and it was acknowledged that injuries were missed in the clinical CT scans when compared to the post-mortem imaging [19].

It must be noted that there is no ‘duplicate publication’ bias between the two studies published by Sanchez et al., as the data collection periods do not overlap [26, 27].

This systematic review may have a language bias, as three full-texts that were not published in English were considered on a case-by-case basis; one was specific to foot and ankle fractures whereas the other two German articles related to trauma imaging. Therefore, they were excluded as they were not deemed to be applicable to SPA imaging.

## Discussion

As a meta-analysis could not be performed due to insufficient eligible studies, a narrative synthesis was performed.

### General interpretation of the results

The lack of diagnostic accuracy data shows the value of reporting guidelines for diagnostic studies, e.g. STARD. One author attempted to report the total accuracy of fracture detection on X-rays and quoted a PPV of 83% [27]. Whilst PPV shows the proportion of positive cases giving positive results, it should not be reported in isolation and should also include the negative predictive value (NPV), which shows the proportion of negative cases giving negative results. Both the PPV and NPV are calculated from the number of True Positives, True Negatives, False Positives and False Negatives. As this level of detail has not been reported in any of the three studies, none of these can be defined as Diagnostic Accuracy Tests. In order to determine the accuracy of a diagnostic test, the ability of the test to detect a medical condition when present and equally to detect the absence of the medical condition when not present is key. Given the number of criteria that are “not reported,” it is not possible to compare the accuracy of LDCT to the current ‘gold standard’ of X-rays when the details have not been reported by the authors.

Radiation dose and image quality are inversely proportional. Due to the risks associated with the use of ionising radiation, especially in children, radiographers aim to acquire diagnostic images using the lowest radiation dose achievable (i.e. the ALARA Principle). However, the lower the radiation dose, the more noise on the image making it more difficult to detect subtle fractures, thereby potentially ‘missing’ or not seeing them. This can have significant implication for the management of the patient. Table 4 compares the radiation doses between the X-ray studies and the LDCT studies with all three studies reporting effective doses for both X-ray and LDCT. Whilst the effective doses can’t be directly compared as each study scanned different body regions, the LDCT scan doses quoted by Lawson et al. for a ‘whole body’ CT scan (Vertex to toes: 0.73 to 1.46) are much lower than the dose for a standard trauma CT scan quoted by Mortiz et al. as being 4.97 mSv [19, 28]. It must be acknowledged that whilst low doses were achieved in these studies, they are of no clinical value if the CT images acquired are not of diagnostic value.

### Limitations of the evidence included

The lack of an international definition of LDCT, other than that generally accepted within the medical imaging industry of less than 1 mSv, is acknowledged. All three studies were case series. It is accepted that these are at the lower levels in the hierarchies of evidence but this does not mean that their evidence should be discarded [29]. Some of the advantages include the fact that they are “easy to do” and “allow detailed investigation into situations which would be unethical or impractical to perform using another study design [30, 31]. The common disadvantages include increased risk of “bias,” they are “difficult to replicate” and “can’t always be generalised to the broader population” [30, 31].

### Implications for practice, policy and future research

One of the strengths of this review is the systematic methodology applied and the fact that grey literature sources were searched.

None of the articles considered the time savings in image acquisition offered by LDCT compared to the Skeletal Survey X-ray series nor the challenges associated with reviewing a whole-body CT dataset. However, this is a potential opportunity for artificial intelligence (AI) to be applied as a screening tool to support the radiologist in guiding them to areas of key interest. Whilst the potential benefits associated with performing a whole-body LDCT skeletal survey compared to a series of X-rays in children are acknowledged, it would be challenging to gain Ethical Approval to determine how low the CT scan radiation dose could be due to the risks associated with the use of ionising radiation. The key risk factor to be considered by the Human Research Ethics Committees is the exposure of young children to ionising radiation. It is noted that Lawson et al. gained retrospective ethical approval for their case series. Further research involving a phantom study will be used to inform future research consisting of a post-mortem study with ethical approval already granted.

The systematic review protocol was submitted for registration with PROSPERO on 23/03/2022: CRD42022276786 [32].

## Conclusion

This systematic review has highlighted the gap in literature to evaluate the effectiveness of LDCT to detect subtle fractures associated with paediatric SPA. Whilst the potential benefits associated with performing a whole-body LDCT skeletal survey compared to a series of X-rays in children are acknowledged, so are the challenges faced by an Ethics Committee in granting approval for such a research study. Therefore, a phantom study to inform a subsequent pilot post-mortem CT study is recommended as the primary investigative methods to assess the ability of low-dose CT to identify subtle fractures and to calculate how low the achievable CT dose can be. A multi-centre study may then be appropriate.

### Supplementary information

Supplementary file 1 (DOCX 27 kb)

## Data Availability

Data generated or analysed during the study are available from the corresponding author by request.

## Abbreviations

CT: Computed tomography
LDCT: Low dose computed tomography
SPA: Suspected physical abuse

## Declarations

### Clinical trial number

Not applicable, as this is a Systematic Literature Review.
