# Supplementary material for: Effectiveness of low dose computed tomography to detect fractures in paediatric suspected physical abuse: a systematic review
Source: Int J Legal Med. 2024 Apr 15;138(5):1857–66. doi: 10.1007/s00414-024-03214-2 (PMC11306378; doi:10.1007/s00414-024-03214-2)
Supplement: Supplementary file 1 — (DOCX 27 kb) [file 414_2024_3214_MOESM1_ESM.docx]

# Supplemental Material

# Appendix A:

# Record of Grey Literature sources searched

## Targeted Website Browsing/Searching

| **Date** | **Organisation name** | **URL** | **# of items screened (uploaded to citation management software)** |
| --- | --- | --- | --- |
| 20/6/21 | The Royal College of Radiologists (UK) | <https://www.rcr.ac.uk/> | “Physical abuse”  = 50 results 1 uploaded = [The radiological investigation of suspected physical abuse in children - BFCR(17)4](https://www.rcr.ac.uk/publication/radiological-investigation-suspected-physical-abuse-children) |
| 20/6/21 | RANZCR | <https://www.ranzcr.com/> | “non-accidental injury” = 2 results. 0 uploaded.  “non accidental injury” = 58 results. 3 screened:   - The use of Diagnostic Imaging for Screening purposes and non-referred investigations (2005) - Development of the Faculty of Post-mortem Imaging (2018) - Recommendations for Imaging in Children in Non-Dedicated Pediatric Centres (2021)   0 uploaded. |
| 20/6/21 | ACR | <https://www.acr.org/>  <https://www.acr.org/Clinical-Resources/ACR-Appropriateness-Criteria> | “Physical abuse”  = 1 result 0 uploaded <https://www.acr.org/Clinical-Resources/ACR-Appropriateness-Criteria>  “Suspected physical abuse”  = 1 result containing 4 documents 1 document (narrative) uploaded: https://acsearch.acr.org/docs/69443/Narrative/ https://acsearch.acr.org/docs/69443/LitSearch/ |
| 20/6/21 | ESPR | <https://www.espr.org/> | “Child abuse”  = 8 results  0 uploaded |
| 20/6/21 | ISFRI | <http://www.isfri.org/> | No search function. 0 results under ‘Pediatric Imaging’ webpage |
| 20/6/21 | WHO | <https://www.who.int/> | “Child abuse”  = 5 results  0 uploaded |

## Grey Literature Database Search

| **Date** | **Database name & URL** | **Search strategy(s)/ words searched including (if applicable) how items were selected*.*** | **# of items retrieved/ search results** | **# of items screened (uploaded to citation management software)** |
| --- | --- | --- | --- | --- |
| 15/5/2022 | NICE  <https://www.nice.org.uk/> | “Child abuse”  All results retrieved were screened for relevance by 1 reviewer | 46 | 0 |
| 15/5/2022 | ProQuest Dissertations  <https://www-proquest-com.ezproxy.lib.monash.edu.au/advanced> | (child abuse OR non accidental injury OR physical abuse) AND child AND (computed tomography) NOT (head OR cranial)  limited to ‘2011-01-01 to 2022-04-30” | 58 | Results (1,2,16,20,21) already included. |
| 15/5/2022 | NHMRC  <https://g-i-n.net/international-guidelines-library/> | child abuse  suspected physical abuse  non accidental injury | 6  0  0 | 0  0  0 |

## Search Engine Searching

| **Date** | **Search engine** | **Search strategy(s) including how items were selected** | **# of items screened (uploaded to citation management software)** |
| --- | --- | --- | --- |
| 15/5/2022 | Google  <https://www.google.com> | (child abuse OR non-accidental injury OR physical abuse) AND (child) AND (CT OR computed tomography) -------------------------------  Items were selected by scanning the first 10 pages of results | About 1,860,000 results (0.71 seconds)  1 uploaded |
| 15/5/2022 | Google Advanced  <https://www.google.com.au/advanced_search> | (child abuse OR non-accidental injury OR physical abuse) AND (child) AND (CT OR computed tomography)  --------------------------------  (CT OR computed tomography) AND (child abuse OR non-accidental injury OR physical abuse) AND (child)  -------------------------------  Items were selected by scanning the first 10 pages of results from each search | About 2,540,000 results (0.49 seconds)  0 uploaded  -----------------------------  About 1,080,000 results (0.65 seconds)  5 uploaded |
| 15/5/2022 | Google Scholar  <https://scholar.google.com.au/> | (child abuse OR non-accidental injury OR physical abuse) AND (child) AND (CT OR computed tomography)  -------------------------------  Items were selected by scanning the first 10 pages of results | About 53,100 results (0.11 sec)  0 uploaded |
| 15/5/2022 | DuckDuckGo  [https://duckduckgo.com/](https://duckduckgo.com/?q=(child+abuse+OR+non-accidental+injury+OR+physical+abuse)+AND+(child)+AND+(CT+OR+computed+tomography)+&t=h_&ia=web) | (child+abuse+OR+non-accidental+injury+OR+physical+abuse)+AND+(child)+AND+(CT+OR+computed+tomography)  -------------------------------  Items were selected by scanning the first 100 results | Number of results not reported  0 uploaded |
| 15/5/2022 | Trip  <https://www.tripdatabase.com/> | PICO search  POPULATION Children  INTERVENTION computed tomography OR ct  COMPARISON X-ray  OUTCOME child abuse OR non accidental injury OR suspected physical abuse  -------------------------------  Items were selected by scanning all 87 results | 87 results  0 uploaded |
| 15/5/2022 | Millionshort  <https://millionshort.com/> | (CT OR computed tomography) AND (child abuse OR non-accidental injury OR physical abuse) AND (child)  -------------------------------  Items were selected by scanning the first 10 pages of results | 5,230,000 results found (0.573 seconds)  0 uploaded |

# Appendix B:

# Search strategies for peer-reviewed literature

Ovid MEDLINE(R) and Epub Ahead of Print, In-Process, In-Data-Review & Other Non-Indexed Citations, Daily and Versions <1946 to May 13, 2022>

1 Tomography, X-Ray Computed/ 408598

2 Compute* tomography.mp. 353247

3 CT.mp. 398923

4 Child/ 1841658

5 children.mp. 1150851

6 child.mp. 2283476

7 Child, Preschool/ 976011

8 Infant/ 846493

9 Pediatrics/ 57474

10 pediatric.mp. 69333

11 Infant, Newborn/ 649394

12 4 or 5 or 6 or 7 or 8 or 9 or 10 or 11 3058809

13 Child Abuse/ 24549

14 Fractures, Bone/ 68289

15 "Wounds and Injuries"/ 80730

16 Non-accidental injury.mp. 466

17 Non-accidental trauma.mp. 174

18 (child adj2 abuse).mp. 38401

19 (physical adj2 abuse).mp. 7834

20 Physical Abuse/ 1022

21 maltreatment.mp. 9290

22 Physical child abuse.mp. 273

23 shaken baby*.mp. 972

24 suspected physical abuse/ 0

25 Shaken Baby Syndrome/ 652

26 13 or 14 or 15 or 16 or 17 or 18 or 19 or 20 or 21 or 22 or 23 or 24 or 25 190669

27 low dose.mp. 108751

28 1 or 2 or 3 or 27 874384

29 12 and 26 and 28 1565

30 limit 29 to yr="2011 -Current" 750

31 (head or skull or cereb* or crani*).mp. 1427817

32 30 not 31 456

33 202111*.dt,ez,da. 246280

34 32 and 33 7

## Cochrane Library

"Radiation Protection" OR "Radiation Exposure" OR "Radiation Dosage" OR "Radiation Monitoring" OR "radiation dosage*" OR "radiation dose*" in Title Abstract Keyword AND "Tomography, X-Ray Computed" OR "Compute* NEXT tomography" OR CT OR "low dose" in Title Abstract Keyword AND Child OR children OR child OR Child, Preschool OR Infant OR Pediatrics OR pediatric OR Infant, Newborn in Title Abstract Keyword AND "Child Abuse" OR "Fractures, Bone" OR "Wounds and Injuries" OR "Non-accidental injury" OR "Non-accidental trauma" OR "child NEAR/2 abuse" OR "physical NEAR/2 abuse" OR "Physical Abuse" OR maltreatment OR "Physical child abuse" OR shaken NEXT baby* OR "suspected physical abuse" OR "Shaken Baby Syndrome" in Title Abstract Keyword - (Word variations have been searched)

## Scopus

## Scopus via Elsevier

## ( ( INDEXTERMS ( "Tomography, X-Ray Computed" )  OR  TITLE-ABS-KEY ( "Compute* tomography" )  OR  TITLE-ABS-KEY ( ct )  OR  TITLE-ABS-KEY ( "low dose" ) )  AND  ( INDEXTERMS ( child )  OR  TITLE-ABS-KEY ( children )  OR  TITLE-ABS-KEY ( child )  OR  INDEXTERMS ( "Child, Preschool" )  OR  INDEXTERMS ( infant )  OR  INDEXTERMS ( pediatrics )  OR  TITLE-ABS-KEY ( pediatric )  OR  INDEXTERMS ( "Infant, Newborn" ) )  AND  ( INDEXTERMS ( "Child Abuse" )  OR  INDEXTERMS ( "Fractures, Bone" )  OR  INDEXTERMS ( "Wounds and Injuries" )  OR  TITLE-ABS-KEY ( "Non-accidental injury" )  OR  TITLE-ABS-KEY ( "Non-accidental trauma" )  OR  TITLE-ABS-KEY ( child  W/2  abuse )  OR  TITLE-ABS-KEY ( physical  W/2  abuse )  OR  INDEXTERMS ( "Physical Abuse" )  OR  TITLE-ABS-KEY ( maltreatment )  OR  TITLE-ABS-KEY ( "Physical child abuse" )  OR  TITLE-ABS-KEY ( "shaken baby*" )  OR  INDEXTERMS ( "suspected physical abuse" )  OR  INDEXTERMS ( "Shaken Baby Syndrome" ) ) )  AND NOT  ( TITLE-ABS-KEY ( head  OR  skull  OR  cereb*  OR  crani* ) )   AND  ( LIMIT-TO ( PUBYEAR ,  2022 )  OR  LIMIT-TO ( PUBYEAR ,  2021 )  OR  LIMIT-TO ( PUBYEAR ,  2020 )  OR  LIMIT-TO ( PUBYEAR ,  2019 )  OR  LIMIT-TO ( PUBYEAR ,  2018 )  OR  LIMIT-TO ( PUBYEAR ,  2017 )  OR  LIMIT-TO ( PUBYEAR ,  2016 )  OR  LIMIT-TO ( PUBYEAR ,  2015 )  OR  LIMIT-TO ( PUBYEAR ,  2014 )  OR  LIMIT-TO ( PUBYEAR ,  2013 )  OR  LIMIT-TO ( PUBYEAR ,  2012 )  OR  LIMIT-TO ( PUBYEAR ,  2011 ) )

## AND  ORIG-LOAD-DATE  >  20211111

## Embase via Ovid

Embase Classic+Embase <1947 to 2022 May 13>

1 "Tomography, X-Ray Computed"/ or "Compute* tomography".mp. or CT.mp. or "low dose".mp. 1800206

2 Child/ or children.mp. or child.mp. or "Child, Preschool"/ or Infant/ or Pediatrics/ or pediatric.mp. or "Infant, Newborn"/ 3768152

3 "Child Abuse"/ or "Fractures, Bone"/ or "Wounds and Injuries"/ or "Non-accidental injury".mp. or "Non-accidental trauma".mp. or (child adj2 abuse).mp. or (physical adj2 abuse).mp. or "Physical Abuse"/ or maltreatment.mp. or "Physical child abuse".mp. or "shaken baby*".mp. or "suspected physical abuse".mp. or "Shaken Baby Syndrome"/ 291976

4 1 and 2 and 3 2012

5 limit 4 to yr="2011 -current" 1155

6 (head or skull or cereb* or crani*).mp. 2062571

7 5 not 6 688

8 limit 7 to dd=20211111-20220430 16

## Web of Science Core Collection

ALL="Tomography, X-Ray Computed" OR ALL="Compute* tomography" OR ALL=CT OR ALL="low dose"

ALL=Child OR ALL=children OR ALL=child OR ALL="Child, Preschool" OR ALL=Infant OR ALL=Pediatrics OR ALL=pediatric OR ALL="Infant, Newborn"

ALL="Child Abuse" OR ALL="Fractures, Bone" OR ALL="Wounds and Injuries" OR ALL="Non-accidental injury" OR ALL="Non-accidental trauma" OR TI=(child NEAR abuse) OR TI=(physical NEAR abuse) OR ALL="Physical Abuse" OR ALL=maltreatment OR ALL="Physical child abuse" OR ALL="shaken baby*" OR ALL="suspected physical abuse" OR ALL="Shaken Baby Syndrome"

#1 AND #2 AND #3

ALL=head OR ALL=skull OR ALL=cereb* OR ALL=crani*

#4 NOT #5
